# Prevalence of Sleep Problems and Its Association With Preterm Birth Among Kindergarten Children in a Rural Area of Shanghai, China

**DOI:** 10.3389/fped.2022.863241

**Published:** 2022-04-25

**Authors:** Ruiping Wang, Jun Chen, Liqun Tao, Yan Qiang, Qing Yang, Bin Li

**Affiliations:** ^1^Clinical Research Center, Shanghai Skin Diseases Hospital, Tongji University, Shanghai, China; ^2^Clinical Research Center, Shanghai Eye Disease Hospital, Shanghai, China; ^3^Lishui Hospital of Traditional Chinese Medicine, Lishui, China; ^4^Songjiang Maternal and Child Health-Care Hospital, Shanghai, China

**Keywords:** kindergarten children, preterm birth, insufficient sleep, sleep problems, association, bedtime delay

## Abstract

**Introduction:**

Healthy sleep in children is critical for their physical and mental health. Although growing evidence indicates the linkage between preterm birth and neural network that regulates sleep architecture, findings on the association between preterm birth and sleep problems among children are still contradictory. In this study, we aimed to understand the prevalence of sleep problems in children aged 3–6 years and to explore the association between sleep problems and preterm birth among children in Shanghai, China.

**Methods:**

We selected 8,586 kindergarten children aged 3–6 years and their mothers in a rural area of Shanghai. Data were collected by questionnaire interview among mothers with informed consent that was signed ahead. Six types of sleep problems (i.e., insufficient sleep, sleepwalk, nightmare, snore, grind teeth, and cry in sleep) were selected in this study. SAS 9.4 was used for data analysis, and *p* < 0.05 was considered statistically significant.

**Results:**

In this study, the prevalence of preterm birth was 9.88% (848/8,586), with a higher prevalence in boys (10.62%) than girls (9.01%). The prevalence of sleep problems was 89.81% among kindergarten children, with 62.50% for snore, 50.35% for grind teeth, 49.20% for cry in sleep, 41.18% for nightmare, 11.67% for insufficient sleep, and 4.44% for sleepwalk. The age of children, family income, and mother's education were associated with the prevalence of sleep problems in children. Logistic regression indicated that sleep problems in preterm children were comparable with the full-term children [odds ratio = 1.13, 95% confidence interval (0.89–1.45)].

**Conclusion:**

Sleep problems were prevalent among children aged 3–6 years in the rural area of Shanghai, and preterm birth was not associated with sleep problems in kindergarten children. We recommend that parents should create limit setting in the home, cultivate similar child-rearing attitudes and beliefs among family members, and encourage children to go to bed earlier.

## Introduction

Sleep is an indispensable and fundamental daily activity that is essential for optimal health in children (1). Sleep has physiological, psychological, and social dimensions, which affects the quality and health of children and adolescents (2, 3). Healthy sleep requires adequate duration, appropriate timing, good quality, regularity, and without sleep disturbance or disorders (1), and healthy sleep in children is critical for their general health, physical development, learning ability, behavior regulation, emotional state, and the stability of mood (4). Sleeping, the number of recommended hours on a regular basis with high quality and without disturbance, is associated with better health outcomes, but insufficient sleep increases the risk of learning problems, injuries, obesity, diabetes, and depression, especially among infants and school-aged children, which is a worldwide public health problem (1, 5).

Sleep is essential for the sustainment of health and homeostatic functions (6). Understanding the importance of sleep for health and wellbeing in the longer term is critical but complicated due to the multiplicity of effects of sleep loss on mood, cognitive function, and physiological systems (6, 7). Sleeping problems, which cover dyssomnias, parasomnias, sleep disorders associated with medical psychiatric disorders, and proposed sleep disorders, may affect many aspects of daily life in a long term, including job performance, mood state, social functioning, and physical and mental health (7). Sleep deficiency is associated with changes in metabolic, inflammatory, and autonomic markers associated with risk for disease. In addition, sleep disruption and sleep deficits lead to mood instability, lack of positive outlook, and impaired neurobehavioral functioning as well. On a population level, insufficient sleep is associated with an increased risk for hypertension and diabetes (8).

Sleep problems are common in all stages of children, including infant, toddler, preschool and school children, and adolescents (9). Sleep in children has been paid much more attention due to the essential stage of physical and mental development. Sleep-related problems including insufficient sleep and disturbed sleep are important parts of the developmental process, which are common among infants and children (10). Operto et al. (11) reported that sleep-related breathing disorders in children refer to several nocturnal events ranging from habitual snoring to obstructive sleep apnea syndrome (OSAS) that may severely impact emotional intelligence, cardiovascular functions, orofacial thrive, and the neuroendocrine and central nervous system. Previous studies indicated that the prevalence of insufficient sleep in children displayed an upward tendency with the increasing age, approximately 10% of children aged 1–3 years have sleep problems (12), and 15–30% of preschoolers have insufficient sleep (13). Meanwhile, disturbed sleep is also prevalent and affects up to 20–30% of children and adolescents (14). Sleep problems in children result from a complex interplay of biological, psychological, developmental, and social influences from a child's family, environment, and culture (15). In recent years, increasing evidence indicates that the reduced quality of sleep or sleep deprivation has negative effects on children's physical and mental development, such as cognitive function, emotional regulation, academic and social performance, and physical health (16), which affects children both in the short and long term.

The Children's Sleep Habit Questionnaire (CSHQ) is widely used as a parent-reported questionnaire to screen for sleep problems in children (17). The CSHQ focuses on sleep disorders commonly in three domains, namely, dyssomnias (difficulty in getting to sleep or stay asleep), parasomnias (sleepwalk, nightmare, night terrors (cry in sleep), bedwetting, restless leg syndrome, and teeth grind), and sleep-disordered breathing (snore in sleep) (9). In recent year, CSHQ is widely used to examine sleep behavior in ordinary children (18, 19) and children with neurodevelopmental disorders as well (20). Ferit et al. (9) used the CSHQ to learn the sleep problems between term and preterm born children and identified that the score of CSHQ subscales, such as bedtime resistance, sleep anxiety, parasomnia, and sleep-disordered breathing, were similar between the groups.

Preterm birth is defined as birth before 37 gestational weeks and has been associated with a variety of adverse neurological outcomes (21). Although growing evidence indicates that preterm birth is linked with the neural network that regulates sleep architecture, the effects of preterm birth on postnatal sleep among preschool children have not been well studied (22). Findings on the association between preterm birth and sleep problems are contradictory (23). A systematic review indicates that prematurity is associated with earlier bedtimes, a lower sleep quality, and more nocturnal awakenings (21). Maria et al. (24) also stated in a review article that preterm infants exhibit irregular sleep schedules, increase motor activity during sleep, and reduce sleep duration and lower arousal threshold when compared with full-term infants. In contrast, Iglowstein et al. (25) has not detected any changes in sleep behaviors including bed-sharing, night awakening, bedtime resistance, and sleep onset difficulties between preterm and full-term children from birth to 10 years of age. So, it is still important to implement studies to clarify the association between preterm birth and sleep problems among children.

Every year, ~15 million infants are born preterm worldwide, which equates to nearly 11% of all live births (26). Although the mortality rates have decreased dramatically with advances in perinatal and neonatal care, preterm infants still face challenges in a neurological and developmental disorder, which is closely related to sleep problems (21). In China, previous studies demonstrate that sleep problem such as insufficient sleep among children is very common (27, 28). In recent years, there are an elevated number of preterm infants since the implementation of the two-child policy and the recently announced three-child policy in China (28). But evidence of the prevalence and sleep problems and their association with preterm birth among children is still limited in China, especially in rural areas. In this study, we investigated the prevalence of sleep problems, including insufficient sleep, snore in night sleep, sleepwalk, cry in sleep, nightmare, and grind teeth in sleep, both among preterm and full-term children aged 3–6 years in a rural area of Shanghai, and tried to explore the association between sleep problems and preterm birth in children so as to provide basic data for future intervention strategy development.

## Methods

### Study Population

We conducted this cross-sectional study during March and September 2018 in the Songjiang district of Shanghai, China. Detailed information regarding sample size estimation and the recruitment process for kindergarten children aged 3–6 years and their parents is available in previously published study (28). In the published study, we focused on the bedtime delay and insufficient sleep among children and explored the potential influencing factors, and we identified that bedtime delay and insufficient sleep in children aged 3–6 years were prevalent, especially in boys and elder children (28). In this study, we included six types of common sleep problems (i.e., insufficient sleep, sleepwalk, nightmare, snore, grind teeth, and cry in sleep) in children to understand their prevalence and the association between sleep problems and preterm birth in this population. Finally, a total of 8,586 questionnaires were completed and included in the final analysis. Songjiang Maternal and Child Health-care Hospital Institution Review Board approved the ethical approval of this study (IRB#20171203). The research coordinators orally communicated with each child and their mothers and then signed the informed consent papers ahead of the questionnaire interview.

### Data Collection

In this study, a questionnaire for data collection was developed by referring to the Children's Sleep Habits Questionnaire (CSHQ), a comprehensive, parent-report sleep screening instrument designed for school-aged children (29). A pilot study indicated that the split-half reliability coefficient (Cronbach's α) of the questionnaire was 0.89, and the content validity coefficient was 0.83. The questionnaire included 3 parts. Part A included 10 demographic questions (age and gender of children, age and education of mother, family yearly income, ethnics, residency status, the only child status of their family). Part B included 12 questions of ordinary sleep habits and sleep problems covering sleep duration, parasomnias, sleep-disordered breathing, and daytime sleepiness (e.g., “when will your child go bed in the night?,” “when will your child fall asleep?,” “when will your child wake up in the morning?,” “how many hours does your child sleep in the daytime?,” “does your child has nightmares?,” “does your child has sleepwalking during night sleep?,” “does your child has night terrors during night sleep?,” “does your child grind his/her teeth in sleep?,” and “does your child snore in night sleep?”). Part C included information for follow-up contact of mother and their children.

### Definition and Index Calculation

In this study, in accordance with the consensus of the American Academy of Sleep Medicine, insufficient sleep among children was defined as those who slept <10 h with the age of 3–5 years and <9 h with the age of 6 years, within each 24 h, respectively (1, 30). Six types of sleep problems (i.e., insufficient sleep, snore in sleep, sleepwalk, cry in sleep, nightmare, and grind teeth in sleep) were formulated according to the common clinical symptom presentations in the *International Classification of Sleep Disorders* (31). In this study, we defined snore in sleep as children with sound due to obstructed air movement during breathing while sleeping; sleepwalk as children with the performance of activities (e.g., talking, sitting up in bed, consuming food, and walking to a bathroom) in a state of low consciousness during sleep; cry in sleep (night terrors) as children who cry in sleep due to the feelings of panic or dread; nightmare as children with the unpleasant dream that with the strong emotional response of fear, despair, anxiety, or great sadness; and grind teeth as children with a grinding or tapping noise during sleep. Sleep problems were rated on a three-point Likert scale (rarely or never = 0–1 night per week, sometimes = 2–4 nights per week, and almost all the time = 5–7 nights per week), and responses were then dichotomized with a sleep problem defined as those who answered “sometimes” or “almost all the time” for each question (28). We calculated the prevalence of sleep problems as the number of children with sleep problems divided by the total number of children. In this study, we recorded parent's education as completed schooling years and classified it into 4 categories including junior high or lower (0–9 years), senior high (10–12 years), college (13–16 years), and postgraduate and above (>16 years). Family yearly income (CNY) was categorized into 5 categories (“ <50,000,” “50,000–10,000,” “100,001–150,000,” “150,001–300,000,” and “>300,000”) with reference to the Chinese household income level in the year of 2018.

### Data Analysis

SAS software (version 9.4) was applied for statistical analysis in this study. Data were described as means as well as standard deviations (SDs) or median as well as the interquartile range (IQR) for quantitative variables, and frequency and prevalence (or percentage) for qualitative variables. Student's *t*-test or Wilcoxon rank-sum test was used to examine the difference in age of children, age of mother, and the total number of sleep problems between preterm children and full-term children. Chi-square test was applied to examine the difference of children's gender, education of mother, family yearly income, ethnics, and residency status of children, the only child of the family, as well as the different prevalence of sleep problems between preterm children and full-term children. Logistic regression was used to calculate the odds ratio (OR) and 95% confidence interval (95% CI) for the different prevalence of sleep problems between preterm children and full-term children with and without the adjustment of potential confounding factors, so as to explore the association between sleep problem and preterm birth in kindergarten children. Figures were produced to show the different prevalence of each sleep problem in kindergarten children with different demographic features and the association between preterm birth and number of sleep problems in children. In this study, the *p*-values < 0.05 (two-tailed) were considered statistically significant.

## Results

In this study, 8,586 children aged 3–6 years in kindergarten, and their mothers were finally analyzed. Notably, 8,586 children included 3,991 girls (46.48%) and 4,595 boys (53.52%), and the average age of children was 4.46 years (SD = 0.96) and that of their mothers was 32.17 years (SD = 3.96), respectively. The majority of mothers had an education of college and above (71.13%), over 40% of children had a family income of over 300,000 CNY per year, 52.94% of children were local residents, and 62.44% of them were the only child of the family (Table 1).

**Table 1:** Demographic feature of kindergarten children and their mothers in a rural area of Shanghai, China.

**Variables**	**Total condition (*n* = 8,586)**	**Birth condition of children**		***χ^2^/t***	***P*** **-value**
		**Preterm children (*n* = 848)**	**Full-term children (*n* = 7,738)**		
Sex of children, *n* (%)				6.143	0.013
Male	4,595 (53.52)	488 (57.55)	4,107 (53.08)		
Female	3,991 (46.48)	360 (42.45)	3,631 (46.92)		
Age of children (years), mean (SD)	4.46 (0.96)	4.54 (0.95)	4.46 (0.96)	2.650	0.008
Age of mother (years), mean (SD)	32.17 (3.96)	32.47 (4.13)	32.14 (3.94)	2.320	0.021
Education of mother, *n* (%)				11.273	0.001
Junior high or under	866 (10.09)	105 (12.38)	761 (9.83)		
Senior high	1,613 (18.79)	182 (21.46)	1,431 (18.49)		
College	5,628 (65.55)	520 (61.32)	5,108 (66.01)		
Postgraduate and above	479 (5.58)	41 (4.83)	438 (5.66)		
Family yearly income (CNY), *n* (%)				10.739	0.001
<50, 000	1,344 (15.65)	172 (20.28)	1,172 (15.15)		
50, 000–100, 000	1,206 (14.05)	124 (14.62)	1,082 (13.98)		
100, 001–150, 000	1,117 (13.01)	97 (11.44)	1,020 (13.18)		
150, 001–300, 000	1,169 (13.62)	109 (12.85)	1,060 (13.70)		
Over 300, 000	3,750 (43.68)	346 (40.80)	3,404 (43.99)		
Ethnics, *n* (%)				0.597	0.440
Han	8,348 (97.23)	828 (97.64)	7,520 (97.18)		
Minority	238 (2.77)	20 (2.36)	218 (2.82)		
Residency status, *n* (%)				0.771	0.380
Local resident	4,545 (52.94)	461 (54.36)	4,084 (52.78)		
Non-local resident	4,041 (47.06)	387 (45.64)	3,654 (47.22)		
The only child of family, *n* (%)				0.501	0.479
Yes	5,361 (62.44)	520 (61.32)	4,841 (62.56)		
No	3,225 (37.56)	328 (38.68)	2,897 (37.44)		

### The Prevalence of Preterm Birth Among Kindergarten Children

In this study, 848 children were born with a gestational week <37, and the prevalence of preterm birth was 9.88% (848/8,586). The prevalence of preterm birth was higher in boys (10.62%, 488/4,595) than in girls (9.01%, 360/3,996), but without statistical significance. The age of preterm children and their mothers was both slightly older than full-term children and their mothers, and differences were all statistically significant. Meanwhile, the proportion of college and above education among mothers of preterm children (66.15%) was statistically lower than mothers of full-term children (71.67%), the family yearly income in preterm children was also lower than in full-term children, and the difference was statistically significant (Table 1).

### The Prevalence of Sleep Problems Among Kindergarten Children

In this study, the median number of sleep problems in children aged 3–6 years was 2 (IQR: 1–3) both for preterm children and full-term children. In this study, 7,711 out of the 8,586 kindergarten children had at least one type of the 6 sleep problems, and the prevalence of any sleep problems was 89.81% in these children, with 11.67% for insufficient sleep, 62.50% for snore in night sleep, 4.44% for sleepwalk, 49.20% for cry in sleep, 41.18% for nightmare, and 50.35% for grind in sleep. The prevalence of sleepwalk in preterm children (6.96%) was higher than in full-term children (4.16%), and the difference was statistically significant (*p* < 0.001). Table 2 indicates that the prevalence of insufficient sleep, snore in night sleep, and nightmare was slightly higher among preterm children, whereas the prevalence of cry in sleep and grind teeth in sleep was lower among preterm children, but without statistically significant (Table 2).

**Table 2:** The sleep problems of kindergarten children aged 3–6 years in a rural area of Shanghai, China.

**Variables**	**Total condition (*n* = 8,586)**	**Birth condition of children**		***χ^2^***	***p*** **-value**
		**Preterm children (*n* = 848)**	**Full-term children (*n* = 7,738)**		
Insufficient sleep, *n* (%)				0.117	0.732
Yes	1,002 (11.67)	102 (12.03)	900 (11.63)		
No	7,584 (88.33)	746 (87.97)	6,838 (88.37)		
Snore in night sleep, *n* (%)				2.238	0.135
Yes	5,366 (62.50)	550 (64.86)	4,816 (62.24)		
No	3,220 (37.50)	298 (35.14)	2,922 (37.76)		
Sleepwalk, *n* (%)				14.090	<0.001
Yes	381 (4.44)	59 (6.96)	322 (4.16)		
No	8,205 (95.56)	789 (93.04)	7,416 (95.84)		
Cry in sleep, *n* (%)				0.025	0.874
Yes	4,224 (49.20)	415 (48.94)	3,809 (49.22)		
No	4,362 (50.80)	433 (51.06)	3,929 (50.78)		
Nightmare, *n* (%)				0.123	0.726
Yes	3,536 (41.18)	354 (41.75)	3,182 (41.12)		
No	5,050 (58.82)	494 (58.25)	4,556 (58.88)		
Grind teeth in sleep, *n* (%)				0.629	0.428
Yes	4,323 (50.35)	416 (49.06)	3,907 (50.49)		
No	4,263 (49.65)	432 (50.94)	3,831 (49.51)		
NO. of total sleep problems, median (IQR)	2.00 (1.00–3.00)	2.00 (1.00–3.00)	2.00 (1.00–3.00)	0.567	0.452
Overall sleep problems, *n* (%)				1.013	0.314
Yes	7,711 (89.81)	110 (90.80)	6,941 (89.70)		
No	875 (10.19)	78 (9.20)	797 (10.30)		

*IQR, Interquartile Range (P_25_-P_75_)*.

**Figure 1:**
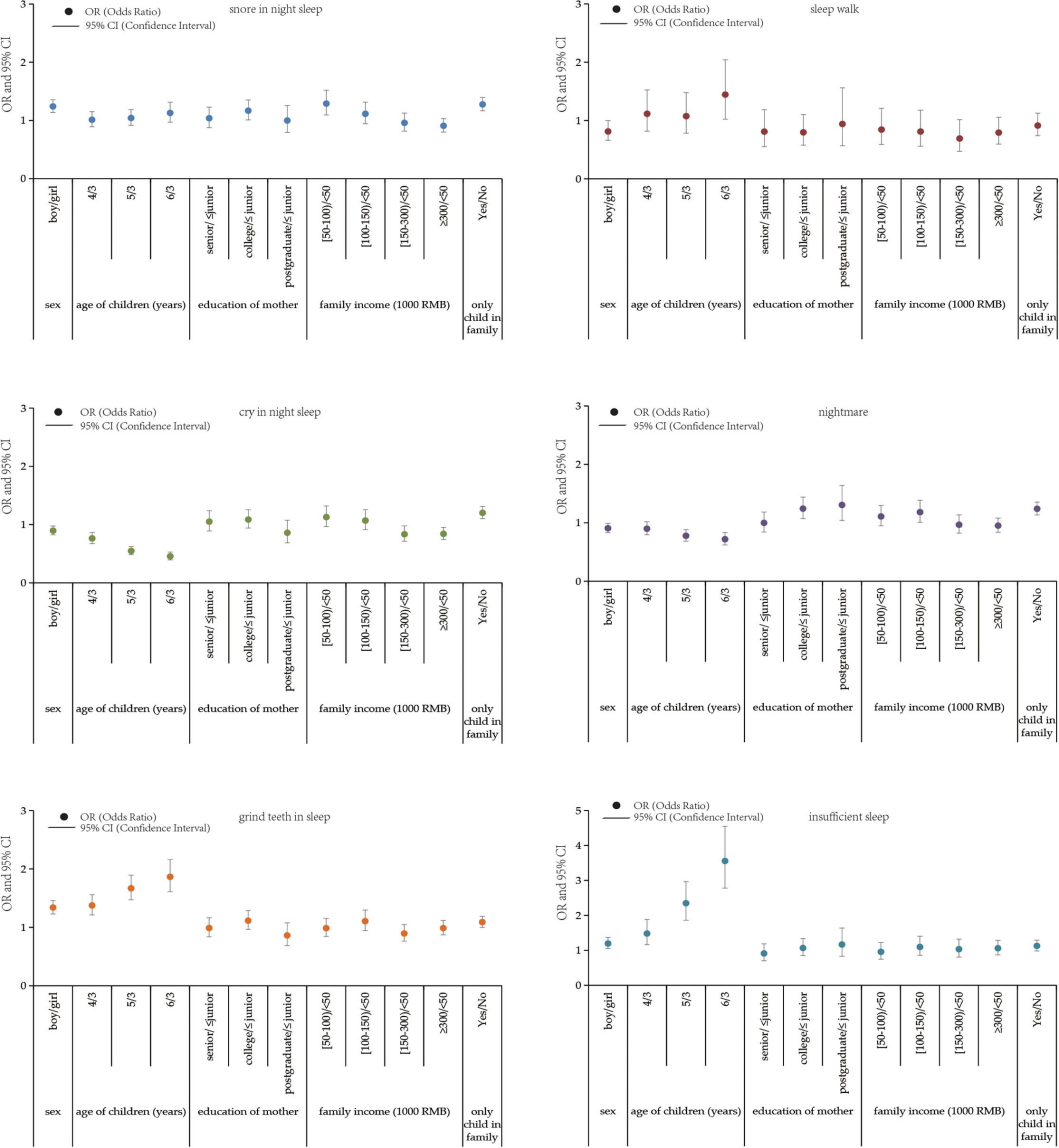
The differences of each sleep problem prevalence among children with different demographic features, including sex, age of children, education of mother, family income, and the only child in the family.

Figure 1 indicates the prevalence of each sleep problems among children with different demographic features. In comparison with girls, boys had a higher prevalence of snore in sleep, grind teeth in sleep, and insufficient sleep. Kindergarten children aged 4–6 years had a higher prevalence of snore in sleep, sleepwalk, grind teeth in sleep, and insufficient sleep, but a lower prevalence of cry in sleep and nightmare than children aged 3 years. In comparison with mothers with junior high and lower education, children whose mothers with postgraduate and above education had a higher prevalence of snore in sleep, nightmare, and insufficient sleep, but a lower prevalence of cry in sleep and grind teeth in sleep. The prevalence of sleep problem decreased slightly with the increase of family yearly income, and children who were the only children of their family had a higher prevalence of sleep problems including snore in sleep, cry in sleep, nightmare, grind teeth, and insufficient sleep.

### The Association Between Preterm Birth and Sleep Problems Among Children

**Figure 2:**
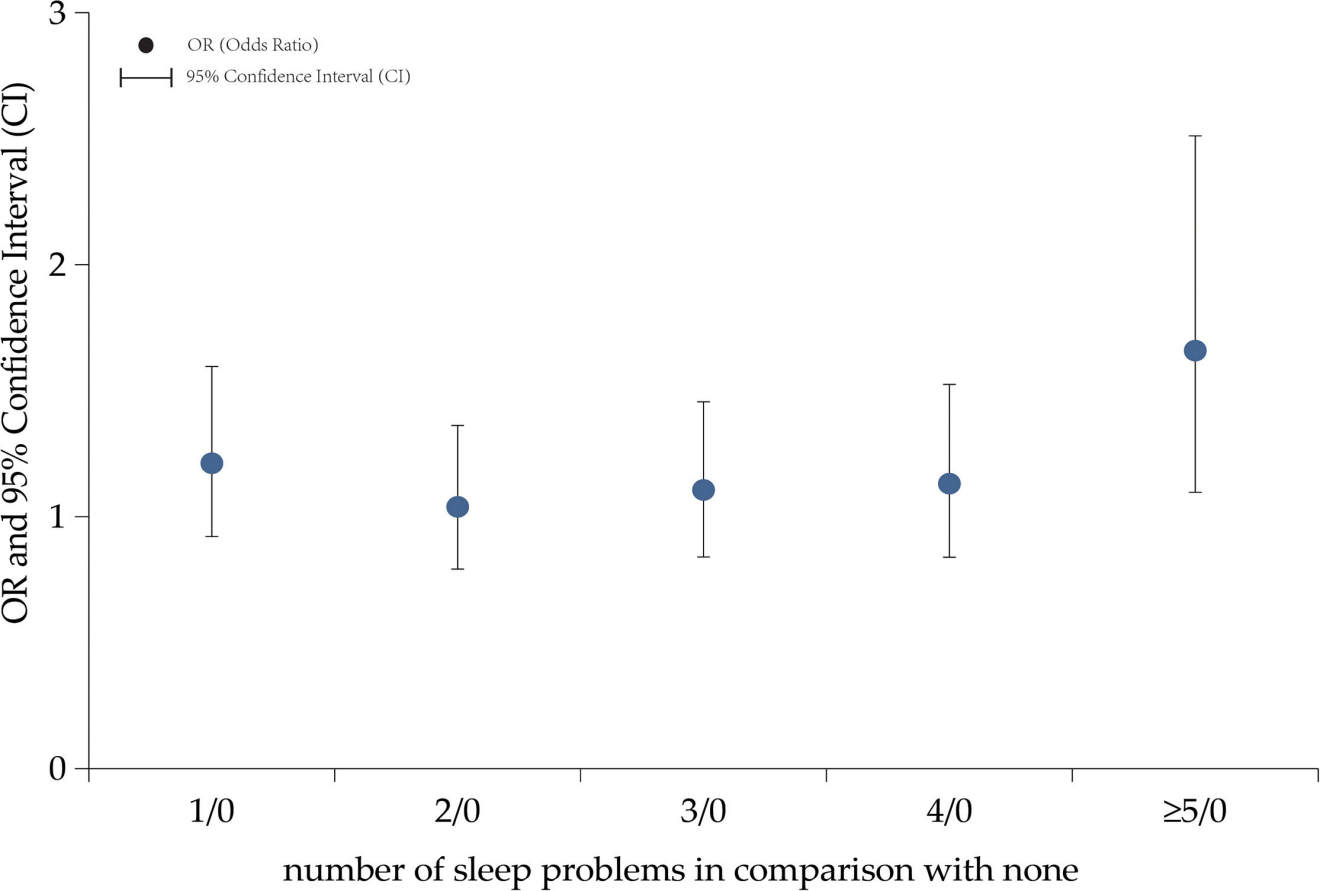
The association between preterm birth and number of sleep problems in kindergarten children aged 3–6 years. In comparison with children without sleep problems, children with sleep problems were more likely to be preterm children, odds ratio (OR) = 1.21 and 95% confidence interval [CI] (0.92–1.60) for 1 sleep problem, OR = 1.04 and 95% CI (0.79-1.36) for 2 sleep problems, OR=1.11 and 95% CI (0.84–1.46) for 3 sleep problems, OR = 1.13 and 95% CI (0.84–1.53) for 4 sleep problems, and OR = 1.66 and 95% CI (1.10–2.51) for 5 sleep problems.

In this study, in comparison with full-term children, preterm children were more likely to have a sleep problem but without statistical significance [OR = 1.13, 95% CI (0.89–1.45)] (Table 3, Figure 2), whereas, preterm children were more likely to have sleepwalk than full-term children [OR = 1.72, 95% CI (1.29–2.29)], even with the adjustment of confounders in Model 2 [OR = 1.73, 95% CI (1.30–2.31)] and in Model 3 [OR = 1.74, 95% CI (1.31–2.32)] (Table 3).

**Table 3:** The association between preterm birth and each sleep problems among kindergarten children aged 3 to 6 years in a rural area of Shanghai, China.

**Sleep problems**	**Model 1**		**Model 2**		**Model 3**	
	**OR**	**95% CI**	**OR**	**95% CI**	**OR**	**95% CI**
**Insufficient sleep**						
Yes	1.04	0.84–1.29	0.99	0.80–1.24	1.01	0.81–1.26
No	1.00		1.00		1.00	
**Snore in night sleep**						
Yes	1.12	0.97–1.30	1.11	0.95–1.28	1.12	0.96–1.29
No	1.00		1.00		1.00	
**Sleepwalk**						
Yes	***1.72***	***1.29–2.29***	***1.73***	***1.30–2.31***	***1.74***	***1.31–2.32***
No	1.00		1.00		1.00	
**Cry in sleep**						
Yes	0.99	0.86–1.14	1.02	0.88–1.18	1.02	0.86–1.18
No	1.00		1.00		1.00	
**Nightmare**						
Yes	1.03	0.89–1.18	1.04	0.90–1.20	1.05	0.91–1.22
No	1.00		1.00		1.00	
**Grind teeth in sleep**						
Yes	0.94	0.82–1.09	0.91	0.79–1.05	0.92	0.80–1.07
No	1.00		1.00		1.00	
**Overall sleep problem**						
At least 1	1.13	0.89–1.45	1.13	0.88–1.44	1.14	0.89–1.46
None	1.00		1.00		1.00	

*Model 1, Crude OR and 95% Confidence Interval (CI) for the association between preterm birth and each sleep problems among kindergarten children aged 3–6 years. Model 2, Adjusted OR and 95% Confidence Interval (CI) for the association between preterm birth and each sleep problems, with the adjustment of children' age and sex. Model 3, Adjusted OR and 95% Confidence Interval (CI) for the association between preterm birth and each sleep problems, with the adjustment of children' age and sex, education of mother, age of mother, and family yearly income. The bold italic values means that the difference was statistically significant*.

Table 3 indicates that the prevalence of insufficient sleep [OR = 1.04, 95% CI (0.84–1.29)], snore in night sleep [OR = 1.12, 95% CI (0.97–1.30)], and nightmare [OR = 1.03, 95% CI (0.89–1.18)] among children with preterm birth was slightly higher than those among children with full-term birth, and the prevalence of grind teeth in sleep [OR = 0.94, 95% CI (0.82–1.09)] among children with preterm birth was slightly lower than children with full-term birth, but none of these differences were statistically significant (*p* > 0.05) (Table 3).

## Discussion

In our previous published study based on the same population, we identified that bedtime delay and insufficient sleep in children aged 3–6 years was prevalent in Shanghai, China (30). Although we have used the same population and included insufficient sleep as well in this study, the purpose was different. In this study, we examined the prevalence of six sleep problems including insufficient sleep, sleepwalk, nightmare, snore in sleep, grind teeth, and cry in sleep and mainly explored its association with preterm birth among children aged 3–6 years in Shanghai, China. Results in this study indicated the following findings: (1) prevalence of the 6 sleep problems ranged from 4.44% (sleepwalk) to 62.50% (snore in sleep) among children aged 3–6 years; (2) boys had a higher prevalence of snore in sleep, grind teeth in sleep, and insufficient sleep than girls. The education of mother was positively associated with the prevalence of insufficient sleep, sleepwalk, and nightmare in children, and family income was negatively associated with the prevalence of all 6 sleep problems in children aged 3–6 years; and (3) except for sleepwalk, preterm birth was not associated with sleep problems in kindergarten children aged 3–6 years.

Previous studies indicate that ~20–61% of preschool children experience sleep problems in the Western population, and the prevalence was higher in Asian countries especially in China (9, 32–35). Previous studies reported 17.1% of sleep problems among infants (32), 20–30% of sleep problems among preschool children (14, 33), and 60.7% of sleep disorders among preschool children in western countries (9). In this study, over 80% of kindergarten children had at least 1 type of sleep problem, and the prevalence was 4.44% for sleepwalk, 11.67% for insufficient sleep, 41.18% for nightmare, 49.20% for cry in sleep, 50.35% for grind teeth in sleep, and 62.50% for snore in night sleep. Findings of sleep problem prevalence among children in this study were in line with previous studies (34, 35). Liu et al. (35) reported that 78.8% of kindergarten children in Chinese urban areas had global sleep disturbance based on CSHQ scores, with 69.5% for bedtime resistance, 56.6% for sleep anxiety, 34.3% for night walk, 19.7% for parosomnias, and 15.1% for insufficient sleep. The discrepancy in sleep problem prevalence among children between western countries and China might be due to cultural, environmental, familial, and individual factors (35, 36). Moreover, sociocultural factors such as sleep spaces, sleep arrangements, school schedules, and academic demands influence the sleep of children *via* numerous pathways, which might also contribute to the discrepancy. Considerable evidence indicates that kindergarten children in China tend to sleep less than their counterparts in western countries, which is mainly due to longer school days and parental expectations regarding the importance of supplemental after school learning opportunities (35, 36). As one important factor for sleep disturbance in children, cosleeping of parents in the first few years was prevalent in China, which might also lead to the discrepancy in the prevalence of sleep problems between western countries and China (35). Thus, a more detailed examination of factors potentially associated with the frequency and severity of sleep problems in children with different sociocultural and familial backgrounds may eventually lead to more appropriate prevention and intervention strategies for sleep problems in children.

Considerable studies demonstrate that sleep disturbance in young children is associated with social demographic factors including sex and age of children, parent's education level, and family income (35, 37, 38). In this study, boys had a higher prevalence of snore in sleep, grind teeth in sleep, and insufficient sleep, which might partially be due to that boys are more physically active and spend more screen time than girls (39, 40). Kindergarten children aged 4–6 years had a higher prevalence of snore in sleep, sleepwalk, grind teeth in sleep, and insufficient sleep than children aged 3 years, and this was in line with previous findings (41). Elder children were more physically active and had a heavier homework load which might attribute to the higher prevalence of insufficient sleep; meanwhile, the increased sleep-related anxiety, concurrent mood, and anxiety disorders among elder children could lead to the prevalence of sleep deprivation as well (32). Moreover, previous studies indicated that children from poorer families had more sleep problems, the prevalence of sleep problem decreased slightly with the increase of family year income that was also identified in this study, and this might be due to that relative income deprivation could generally affect health outcomes and sleep directly by increasing the level of stress and insecurity associated with the awareness of social status (42, 43). In this study, we also noticed that kindergarten children who were the only children of their family had a higher prevalence of sleep problems, and this might be due to that a single child in the family could result in a tendency to over-focus parental attention and diminish limit setting within the family structure, leading to an increased risk of behavior mediated sleep disturbances (28, 35, 44).

This study indicates that the prevalence of preterm birth among kindergarten children was 9.88%, which was in line with findings in previous studies (33). In 2011–2012, 11.5% of births were preterm in Brazil, and a similar one in every ten babies was born prematurely in the United States in 2018 (33, 45). Several risk factors of preterm birth have been identified previously, such as the history of previous spontaneous preterm birth, primiparity, advanced maternal age, smoking, obesity, lower education and family income, and maternal medical conditions (46, 47). Azeez et al. (48) reported a higher proportion of advanced maternal age of mothers with preterm children (30.1%) than those with full-term children (21.7%), and the prevalence of preterm birth among boys (17.0%) was slightly higher than girls (16.6%). In this study, we identified that the prevalence of preterm birth was higher among boys than girls, mothers of preterm children were older than those with full-term birth, the prevalence of preterm birth descended with the increase of family yearly income level, as well as the education level of mothers, and these findings were in line with previous evidence (47–49).

Existing data regarding preterm infant sleeping problems are mixed and discrepancy (33). Preterm birth has been usually associated with increased risk of sleep problems in children, including sleep disturbance induced by chronic asthma and bronchopulmonary dysplasia in preterm children, and obstructive sleep apnea syndrome (OSAS) in preterm children as well, but the evidence is accumulating that preterm infants do not differ in their sleep behavior or problems compared with termed infants (11, 25, 50). In this study, children of preterm birth had a comparable prevalence of sleep problems in comparison with full-term birth children. Similarly, Iglowstein et al. (25) have not detected any changes in sleep duration, night waking, bedtime resistance, and sleep onset difficulties between preterm and termed children. An explanation for comparable sleep patterns and problems between preterm and full-term children was that the maturation of intrinsic sleep-wake mechanism, the developmental process of the child, and parent–child interaction might primarily drive sleep behavior during the childhood, rather than prematurity or neonatal care experience (51, 52). Therefore, taking measures to promote healthy sleep education among parents, creating limit setting in the home, cultivating similar child-rearing attitudes and beliefs in family members, and encouraging children to go to bed earlier is crucial for reducing the prevalence of sleep problems in children (35, 53).

It was interesting that preterm children in this study were more likely to have sleepwalk than full-term birth children, even with the adjustment of confounders. Sleepwalk consists of a series of complex behaviors and arises from the deep non-rapid eye movement sleep (54). Previous studies indicate that sleepwalk declines with age from an estimated prevalence of 3.8–6.5% in children to 1–2.3% in adolescents (53), and the prevalence of sleepwalk in kindergarten children in this study was consistent with the aforementioned studies. The declining trend in sleepwalk mirrors the well-documented reduction in slow-wave sleep across these ages. The slow-wave decline is thought to reflect brain maturation changes, which is consistent with a developmental view of sleepwalk prevalence changes, and is more prevalent among preterm infants (53). This may partially explain the higher prevalence of sleepwalk in children with preterm birth, but the actual association between preterm birth and sleepwalk in children still needs more studies.

This study has some limitations. First, the study design of a cross-sectional study may induce some information bias and only allow the calculation of prevalence. Second, the mother reported information of bedtime at night, the morning wake up time, as well as sleep problems among children might be underreported and lead to a potential risk of underestimation of the prevalence of sleep problems in children. Third, we only included one rural district to implement this investigation due to our limited resources in the year 2018. The sample only from the Songjiang district has limited representatives of the rural areas, even in Shanghai, and we will cooperate with other rural areas in our future studies. Fourth, moreover, since the sleeping issue in children is not only a rural topic, we need to select some children in the urban area in future studies. So the incorporation of some improvements should be considered in the future follow-up studies.

## Conclusion

Sleep problems were prevalent among kindergarten children aged 3–6 years in rural areas of Shanghai, both for preterm and full-term children, and preterm birth was not associated with sleep problems in kindergarten children. We recommend that the health bureau should take measures to promote children's healthy sleep education among parents, and parents should create limit setting in the home for children, cultivate similar child-rearing attitudes and beliefs among their family members, and encourage children to go to bed earlier.

## Data Availability Statement

The raw data supporting the conclusions of this article will be made available by the authors, without undue reservation.

## Ethics Statement

The studies involving human participants were reviewed and approved by Songjiang Maternal and Child Health-Care Hospital Institution Review Board. Written informed consent to participate in this study was provided by the participants' legal guardian/next of kin.

## Author Contributions

RW and BL participated in the study design. RW and JC conducted the study and drafted the manuscript. YQ, LT, and QY participated in the fieldwork. BL revised the manuscript. All authors have read this manuscript and approved the final manuscript.

## Funding

This study was supported by grants from the Shanghai Talent Development Fund (2021073), Intelligence Funds of Shanghai Skin Disease Hospital (2021KYQD01), and the National Key R&D Program of China (2018YFC1705300). The funder had no role in study design, data collection and analysis, decision for publication, or preparation of the manuscript.

## Conflict of Interest

The authors declare that the research was conducted in the absence of any commercial or financial relationships that could be construed as a potential conflict of interest.

## Publisher's Note

All claims expressed in this article are solely those of the authors and do not necessarily represent those of their affiliated organizations, or those of the publisher, the editors and the reviewers. Any product that may be evaluated in this article, or claim that may be made by its manufacturer, is not guaranteed or endorsed by the publisher.

## Abbreviations

CSHQ: Children's Sleep Habit Questionnaire
CHY: Chinese Yuan
SD: Standard Deviation
IQR: Interquartile Range
OR: Odds Ratio
CI: Confidence Interval.

## References

[1] Paruthi S Brooks L D'Ambrosio C Hall WA Kotagal S Lloyd RM. Recommended amount of sleep for pediatric populations: a consensus statement of the american academy of sleep medicine. J Clin Sleep Med. (2016) 12:785–6. 10.5664/jcsm.5866 27250809 PMC4877308

[2] Barclay NL Gregory AM. Quantitative genetic research on sleep: a review of normal sleep, sleep disturbances and associated emotional, behavioral, and health-related difficulties. Sleep Med Rev. (2013) 17:29e40. 10.1016/j.smrv.2012.01.008 22560641

[3] Ozgul B Sebahat C Fahri O. Impact of sleep behaviors on social and emotional problems in three year old children born prematurely. Sleep Med. (2020) 24:173–8. 10.1016/j.sleep.2020.08.004 32858277

[4] McDowall PS Galland BC Campbell AJ Elder DE. Parent knowledge of children's sleep: a systematic review. Sleep Med Rev. (2017) 31:39e47. 10.1016/j.smrv.2016.01.002 26899741

[5] Fitch K Bernstein S Aguilar M Buurnand B Lacalle JR Lazaro P. The Rand/UCLA Appropriateness Method User's Manual. Santa Monica, CA: Rand (2001).

[6] Steiner MA Yanagisawa M Clozel M. The orexin system. basic science and role in sleep pathology. Front Neurol Neurosci. (2021) 45:128–38. 10.1159/isbn.978-3-318-06844-334052807

[7] Jolien V Stein K Jan OC. Effects of psychological and social work factors on self reported sleep disturbance and difficulties initiating sleep. Sleep. (2016) 39:833–46. 10.5665/sleep.5638 26446114 PMC4791617

[8] Hossain JL Shapiro CM. The prevalence, cost implications, and management of sleep sisorders: an overview. Sleep Breath. (2002) 6:85–102. 10.1055/s-2002-32322 12075483

[9] Ferit D Nukhet AC Ulfet VO Ridvan D Betul A. Comparison of sleep problems between term and preterm born preschool children. Sleep Med. (2020) 75:484–90. 10.1016/j.sleep.2020.09.013 33010574

[10] Hysing M Harvey AG Torgersen L Ystrom E Reichborn-Kjennerud T Sivertsen B. Trajectories and predictors of nocturnal awakenings and sleep duration in infants. J Dev Behav Pediatr. (2014) 35:309e16. 10.1097/DBP.0000000000000064 24906032

[11] Operto FF Precenzano F Bitetti I Lanzara V Fontana ML Pastorino GMG. Emotional intelligence in children with severe sleep related breathing disorders. Behav Neurol. (2019) 2019:6530539. 10.1155/2019/6530539 31583023 PMC6748194

[12] Byars K Yolton K Rausch J Lanphear B Beebe D. Prevalence, patterns, and persistence of sleep problems in the first 3 years of life. Pediatrics. (2012) 129:e276–284. 10.1542/peds.2011-0372 22218837 PMC3357046

[13] Leonie FO Julia P Michael S Kathrin H Alexander M Alfred W. Prevalence and course of sleep problems in childhood. Sleep. (2007) 30:1371–7. 10.1093/sleep/30.10.1371 17969471 PMC2266270

[14] Sadeh A Tikotzky L Scher A. Parenting and infant sleep. Sleep Med Rev. (2010) 14:89e96. 10.1016/j.smrv.2009.05.003 19631566

[15] Kim DYD Daniel YTG. Comparison of sleep characteristics, patterns, and problems in young children within the southeast Asian region. Behav Sleep Med. (2017) 2017:1–8. 10.1080/15402002.2017.1342168 28613954

[16] Chorney DB Detweiler MF Morris TL Kuhn BR. The interplay of sleep disturbance, anxiety, and depression in children. J Pediatr Psychol. (2007) 33:339e48. 10.1093/jpepsy/jsm105 17991689

[17] Beth LGJ Stephanie LS Karen TBS Liu J Anders TF. The children's sleep habit questionnaire in toddlers and preschool children. J Dev Behav Pediatr. (2007) 29:82–8. 10.1097/DBP.0b013e318163c39a 18478627

[18] Malow BA Marzec ML Mcgrew SG Wang L Henderson LM Stone WL. Characterizing sleep in children with autism spectrum disorders: a multidimensional approach. Sleep. (2006) 29:1563–71. 10.1093/sleep/29.12.1563 17252887

[19] Seifer R Sameroff A Dickstein S Hayden LC Schiller M. Parental psychopathology and sleep variation in children. Sleep Diseases. (1996) 5:715–27. 10.1016/S1056-4993(18)30358-4

[20] Owens J Maxim R Mcguinn M Nobile C Msall M Alario A. Television viewing habits and sleep disturbances in school-aged children. Pediatrics. (1999) 104:e27. 10.1542/peds.104.3.e27 10469810

[21] Simone SMV Willemijn JM Kervezee L Van DHA Verschuren O Pillen S. The relationship between preterm birth and sleep in children at school age: a systematic review. Sleep Med Rev. (2021) 57:101447. 10.1016/j.smrv.2021.101447 33611088

[22] Bennet L Walker DW Horne RSC. Waking up too early-the consequences of preterm birth on sleep development. J Physiol. (2018) 596:5687–708. 10.1113/JP274950 29691876 PMC6265542

[23] Biggs SN Meltzer LJ Tapia IE Traylor J Nixon GM Horne RS. Sleep/wake patterns and parental perceptions of sleep in children born preterm. J Clin Sleep Med. (2016) 12:711–7. 10.5664/jcsm.5802 26857057 PMC4865558

[24] Maria G Katerina H Evangelos P. Sleep and prematurity: sleep outcomes in preterm children and influencing factors. World J Pediatr. (2019) 15:209–18. 10.1007/s12519-019-00240-8 30830664

[25] Iglowstein I Latal Hajnal B Molinari L Largo RH Jenni OG. Sleep behaviour in preterm children from birth to age 10 years: a longitudinal study. Acta Paediatr. (2006) 95:1691–3. 10.1080/08035250600686938 17129987

[26] Chawanpaiboon S Vogel JP Moller AB Lumbiganon P Petzold M Hogan D. Global, regional, and national estimates of levels of preterm birth in 2014: a systematic review and modelling analysis. Lancet Glob Health. (2019) 7:e37e46. 10.1016/S2214-109X(18)30451-0 30389451 PMC6293055

[27] Wang G Xu G Liu Z Lu N Ma R Zhang E. Sleep patterns and sleep disturbances among Chinese school-aged children: prevalence and associated factors. Sleep Med. (2013) 14:45–52. 10.1016/j.sleep.2012.09.022 23218539

[28] Chen X Qiang Y Liu X Yang Q Zhu Q Li B. The prevalence of insufficient sleep and bedtime delay among kindergarten children aged 3 to 6 years in a rural area of Shanghai: a cross-sectional study. Front Pediatr. (2021) 9:759318. 10.3389/fped.2021.759318 34900866 PMC8655690

[29] Judith AO Anthony S Melissa MG. The children's sleep habit questionnaire (CSHQ): psychometric properties of a survey instrument for school aged children. Sleep. (2000) 23:1–10. 10.1093/sleep/23.8.1d 11145319

[30] Cheng W Rolls E Gong W Du J Zhang J Zhang XY. Sleep duration, brain structure, and psychiatric and cognitive problems in children. Mol Psychiatry. (2020) 26:3992–4003. 10.1038/s41380-020-0663-2 32015467 PMC8855973

[31] International Classification of Sleep Disorders. Diagnostic and coding manual. In: Thorpy MJ, Chairman. Diagnostic Classification Steering Committee. Rochester, MN: American Sleep Disorder Association (1990).

[32] Ariel AW Jodi AM Harriet H Jon Q. Child sleep behaviors and sleep problems from infancy to school age. Sleep Med. (2019) 63:5–8. 10.1016/j.sleep.2019.05.003 31600659 PMC6859188

[33] Sadeh A Mindell JA Luedtke K Wiegand B. Sleep and sleep ecology in the first 3 years: a web-based study. J Sleep Res. (2009) 18:60e73. 10.1111/j.1365-2869.2008.00699.x 19021850

[34] Chou Y. Survey of sleep in infants and young children in northern Taiwan. Sleep Biol Rhythms. (2007) 5:40–9. 10.1111/j.1479-8425.2006.00245.x

[35] Liu ZJ Wang GH Geng Li Luo J Li N Owens J. Sleep patterns, sleep disturbances, and associated factors among Chinese urban kindergarten children. Behav Sleep Med. (2014) 14:100–17. 10.1080/15402002.2014.963581 25396279

[36] Amintehran E Ghalehbaghi B Asghari A Jalilolghadr S Ahmadvand A Foroughi F. High prevalence of sleep problems in school- and preschool-aged children in Tehran: a population based study. Iran J Pediatr. (2013) 23:45–52. 10.1136/edpract-2011-301250 23550175 PMC3574991

[37] Blair PS Humphreys JS Gringras P Taheri S Scott N Emond A. Childhood sleep duration and associated demographic characteristics in an English cohort. Sleep. (2012) 35:353–60. 10.5665/sleep.1694 22379241 PMC3274336

[38] Yang Q Bu Y Dong S Fan S Wang L. A comparison of sleeping problems in school-age children between rural and urban communities in China. J Pediatr Child Health. (2009) 45:414–8. 10.1111/j.1440-1754.2009.01530.x 19712177

[39] Zhang Z Adamo K Ogden N Goldfield GS Okely AD Kuzik N. Longitudinal correlates of sleep duration in young children. Sleep Med. (2021) 78:128–34. 10.1016/j.sleep.2020.12.023 33429288

[40] Guerrero MD Barnes JD Chaput JH Tremblay MS. Screen time and problem behaviors in children: exploring the mediating role of sleep duration. Int J Behav Nutr Phys Act. (2019) 16:105. 10.1186/s12966-019-0862-x 31727084 PMC6854622

[41] Hong Z Wei C Wang RM. The investigation on sleep disturbances for aged 0–5 years children in Fuzhou. Strait J Prev Med. (2007) 13:21–3.

[42] Marco CA Wolfson AR Sparling M Azuaje A. Family socioeconomic status and sleep patterns of young adolescents. Behav Sleep Med. (2011) 10:70–80. 10.1080/15402002.2012.636298 22250780 PMC5875933

[43] Marta B Simone G. Family income and maternal deprivation: do they matter for sleep quality and quantity in early life? evidence from a longitudinal study. Sleep. (2017) 40:1–9. 10.1093/sleep/zsw066 28364413 PMC6410939

[44] Kocevska D Lysen T Dotinga A Koopman-Verhoeff ME Luijk MPCM Antypa N. Sleep characteristics across the lifespan in 1. 1 million people from the Netherlands, United Kingdom and United States: a systematic review and meta-analysis. Nat Hum Behav. (2021) 5:113–22. 10.1038/s41562-020-00965-x 33199855

[45] Lupini F Leichman ES Lee C Mindell JA. Sleep pattern, problems and ecology in young children born preterm and full-term and their mothers. Sleep Med. (2021) 81:443–50. 10.1016/j.sleep.2021.03.011 33839374

[46] Raisanen S Gissler M Saari J Kramer M Heinonen S. Contribution of risk factors to extremely, very and moderately preterm births-register based analysis of 1390742 singleton births. PLoS ONE. (2013) 8:1–7. 10.1371/journal.pone.0060660 23577142 PMC3618176

[47] Lucy TB Daan GN David RH Hein OJ. Association of socioeconomic status and clinical and demographic conditions with the prevalence of preterm birth. Int J Gynaecol Obstet. (2020) 149:359–69. 10.1002/ijgo.13143 32176323 PMC7266696

[48] Azeez B Chinyere E Osayame E Nancy W Jenna L Iretiola F. Characteristics and risk factors of preterm births in a tertiary center of Logos, Nigeria. Pan Afr Med J. (2016) 24:1–8. 10.11604/pamj.2016.24.1.8382 27583065 PMC4992393

[49] Newburn-Cook CV Onyskiw JE. Is older maternal age a risk factor for preterm birth and fetal growth restriction? a systematic review. Health Care Women Int. (2005) 26:852–75. 10.1080/07399330500230912 16214797

[50] Kristine MS Silje KF Jacob G Mjell DH Trond M Mari H. Children born extremely preterm had different sleeping habits at 11 years of age and more childhood sleep problems than term-born children. Acta Paediatr. (2017) 106:1966–72. 10.1111/apa.13991 28714101

[51] Jenni OG Fuhrer HZ Iglowstein I Molinari L Largo RH. A. longitudinal study of bed sharing and sleep problems among Swiss children in the first 10 years of life. Pediatrics. (2005) 115:233–40. 10.1542/peds.2004-0815E 15866857

[52] Jenni OG LeBourgeois MK. Understanding sleep-wake behavior and sleep disorders in children: the value of a model. Curr Opin Psychiatry. (2006) 19:282–7. 10.1097/01.yco.0000218599.32969.03 16612214 PMC2980811

[53] Stallman HM Kohler M Wilson A Biggs S Dollman J Martin J. Self-reported sleepwalking in Australian senior secondary school students. Sleep Med. (2016) 25:1–3. 10.1016/j.sleep.2016.06.024 27823701

[54] Laberge L Tremblay RE Vitaro F Montplaisir J. Development of parasomnias from childhood to eary adolescence. Pediatrics. (2000) 106:67–74. 10.1542/peds.106.1.67 10878151

